# Targeting synovial inflammation in knee osteoarthritis: translational insights for diagnosis and therapy

**DOI:** 10.1016/j.jot.2025.10.004

**Published:** 2026-01-30

**Authors:** Ali Mobasheri, Francisco Castro-Domínguez, Oreste Gualillo, Stefan Kluzek, Silvia Paz Ruiz, Raquel Largo

**Affiliations:** a Research Unit of Health Sciences and Technology, Faculty of Medicine, University of Oulu, Oulu, Finland; b Department of Regenerative Medicine, State Research Institute Centre for Innovative Medicine, Vilnius, Lithuania; c Department of Joint Surgery, First Affiliated Hospital of Sun Yat-sen University, Guangzhou, Guangdong, China; d Faculty of Medicine, Université de Liège, Belgium; e Rheumatology Department, Teknon Medical Center, Quirón Salud Group, Barcelona, Spain; f SERGAS (Servizo Galego de Saúde), Área Sanitaria de Santiago de Compostela e Barbanza, Santiago University Clinical Hospital, Santiago de Compostela, Spain; g IDIS, Instituto de Investigación Sanitaria de Santiago, C027 Group NEIRID, NeuroEndocrine Interactions in Rheumatic and Inflammatory Diseases, Santiago de Compostela, Spain; h Faculty of Medicine and Health Sciences, University of Nottingham, Nottingham, England; i University of Oxford, LMH, Oxford, England; j UK Sports Institute, Bisham Abbey, England; k Terminal 4 Communications, Hilversum, Netherlands; l Joint and Bone Research Unit, IIS-Fundacion Jiménez Diaz UAM, Madrid, Spain

**Keywords:** Osteoarthritis, Knee, Synovitis, Inflammation, Polyacrylamide hydrogel, PAAG, Viscosupplementation

## Abstract

Synovial inflammation is a central feature of knee osteoarthritis (OA), linking systemic and local pathogenic pathways with clinical outcomes. This review synthesises current knowledge on synovial structure and function, and the molecular mechanisms that drive synovitis, including metaflammation, mechanoflammation, mechanotransduction, and immune cell polarisation. It further explores how these processes contribute to structural degeneration, pain, and disease progression. Diagnostic approaches are discussed, with emphasis on biochemical biomarkers, imaging modalities, and the limited reliability of pain alone as a disease marker. By integrating clinical phenotypes with molecular endotypes, the review features how precision medicine can reshape OA care. Therapeutic strategies are presented across the translational spectrum, from oral and intra-articular drugs in clinical use to emerging investigational therapies, multitargeting approaches, and novel gene therapies. This review advocates for phenotype- and endotype-informed interventions as a pathway to more effective and personalised management of synovitis-driven knee OA. The translational value of this work lies in integrating molecular mechanisms with clinical strategies to guide early diagnosis, refine therapeutic targeting, and inform the design of future trials.

## Graphical abstract

Knee OA is a complex and heterogeneous disease involving multiple overlapping pathophysiological mechanisms. Rather than existing in isolation, OA endotypes are interconnected and influence one another. Chronic inflammation may lead to structural damage, while tissue injury and joint degradation can, in turn, exacerbate synovial inflammation. Likewise, metabolic dysregulation sustains low-grade inflammation, and impaired repair capacity may drive fibrotic remodelling. Recognising this interplay between endotypes is essential for identifying appropriate mechanism-based therapeutic strategies and for advancing towards more personalised management of the disease.

**Figure:**
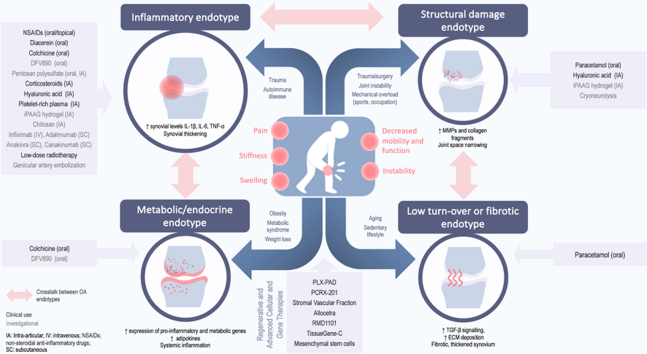

## 1 Introduction

Osteoarthritis (OA) is the most common form of arthritis associated with pain, disability, and loss of function. It encompasses a wide range of aetiologies, from primary degenerative changes to post-traumatic, hereditary, inflammatory, metabolic, and systemic conditions such as rheumatoid arthritis and gout. Knee OA has a global prevalence of 22·9 % in individuals aged 40 and over [1]. With population growth and aging, estimates suggest a 74·9 % increase in knee OA cases by 2050 [2], affecting individuals’ quality of life and imposing a substantial burden on global healthcare systems.

Knee OA is a whole joint disease that entails structural alterations in the hyaline articular cartilage, subchondral bone, ligaments, menisci, capsule, synovium, and muscles surrounding the joint [3]. Clinically, it manifests with considerable variability: some patients experience prominent inflammatory symptoms with joint effusion, while others remain relatively asymptomatic despite structural degeneration. Pain and swelling often fluctuate over time, influenced by mechanical stress, trauma, physical activity, or comorbid conditions [4].

Among the diverse pathophysiological processes contributing to OA, synovial inflammation (synovitis) has emerged as a central driver of symptom burden, structural progression, and treatment response [5]. However, the cause–and–effect relationship between synovitis and joint degeneration remains complex. In some cases, inflammation may be a response to cartilage breakdown or mechanical stress; in others, it may precede and amplify joint damage, particularly in the context of metabolic dysfunction or immune dysregulation [3,5].

Recognising this dynamic interplay has been instrumental in shaping a major shift in our understanding of OA pathophysiology over the past few decades. Rather than being viewed solely as a degenerative condition caused by mechanical wear and tear, OA is now recognised as a complex, multifactorial joint disorder in which chronic inflammation plays a central role [6].

In this narrative review, we provide a comprehensive overview of the role of synovial inflammation, focusing specifically on knee OA. We highlight its translational relevance by linking molecular mechanisms to clinical outcomes and emphasise emerging therapeutic approaches for selective targeting based on distinct inflammatory and metabolic profiles, thereby supporting precision care.

## 2 Methodology

This narrative review provides a comprehensive overview of synovial inflammation in knee OA, with a focus on its molecular characteristics, clinical implications, and emerging therapeutic strategies. We conducted a targeted literature search using PubMed and Scopus databases. Key search terms included “knee osteoarthritis,” “synovial inflammation,” “synovitis,” “molecular pathways,” and “intra-articular therapies.” Studies were selected to address the main domains covered in this review: (1) synovial structure and function; (2) cellular and molecular mechanisms of synovitis and structural damage in knee OA, including metaflammation, mechanoflammation, mechanotransduction of mechanical stress, immune cell polarisation, and downstream tissue degeneration; (3) biochemical, imaging, and clinical markers of knee OA, with a focus on biomarkers and pain as indicators of disease activity; (4) clinical phenotypes and molecular endotypes of OA; (5) therapeutic approaches, encompassing pharmacological and intra-articular treatments in clinical use, investigational therapies targeting mechanoflammation and metaflammation, and multitargeting strategies; and (6) gene therapies, including macrophage-directed cell products, mesenchymal stem cell approaches, and vector-based gene delivery systems. Priority was given to high-quality reviews and meta-analyses, clinical trials, and translational studies published in English between January 2020 and May 2025. Additional seminal or mechanistic studies were included when foundational to understanding current concepts. Expert review and clinical insight guided the selection and synthesis of evidence, with particular attention to inflammatory and metabolic endotypes relevant to precision treatment approaches in knee OA.

## 3 Results

### 3.1 Synovial structure and function

The synovium is a specialised connective tissue that envelops diarthrodial joints, encircles tendons, and forms the lining of bursae and fat pads [7]. It plays a critical role in the anatomy of synovial joints, providing both structural support and functional roles that are vital for joint health and mobility [8]. It serves as a seal for the synovial cavity, preventing fluid from escaping into surrounding tissues.

The synovium typically consists of two layers: the outer subintima and the inner intima. The subintima contains extracellular matrix (ECM) components, such as collagen, adhesins, and fibronectin, as well as resident macrophages, vessels, and a variable number of adipocytes depending on the location [9]. It is rich in type I collagen, well-vascularized with microvascular blood supply, lymphatic vessels, and nerve fibres, but has low cellular content. The intima, adjacent to the joint cavity, is a thin layer of cells mainly composed of type A (macrophage-like) and type B (fibroblast-like) synoviocytes, with the fibroblast-like synoviocytes being the predominant cell type in a healthy synovium. Fibroblast-like synoviocytes produce proteoglycan 4, hyaluronic acid (HA) and lubricin, which contribute to the highly viscous and lubricating properties of synovial fluid [10]. This fluid not only lubricates the joint but also provides a matrix for the delivery of essential nutrients to chondrocytes and the removal of metabolic waste substances, as articular cartilage lacks its own vascular or lymphatic supply, relying on diffusion and gradients provided by the synovial microcirculation. The ECM in the synovium, along with monocytic resident cells, and adipocytes, forms a three-dimensional structure that acts as an internal immune barrier in the synovial lining, helping to limit inflammation [11]. Overall, the unique structure and function of the synovium are essential for maintaining joint integrity and health, facilitating smooth movement, and determining the pathophysiology of OA.

### 3.2 Cellular and molecular mechanisms of synovitis and structural damage in knee OA

**Fig. 1:**
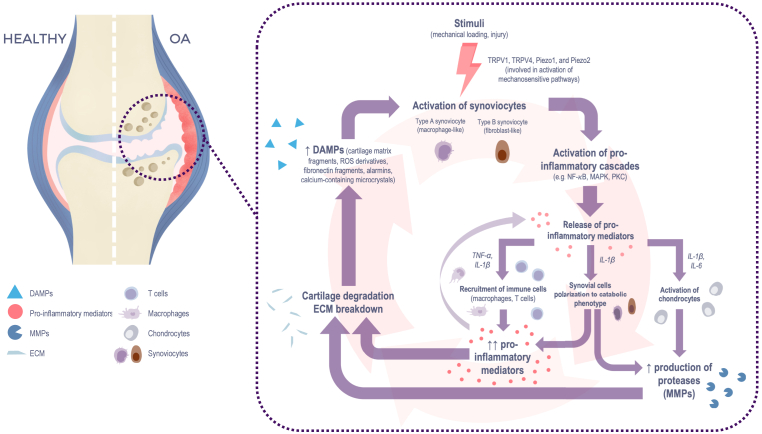
**Key cellular and molecular mechanisms of synovial inflammation-Footnote** This figure illustrates the transition from a healthy knee joint to an osteoarthritic state, emphasising the self-perpetuating vicious cycle of synovial inflammation. Mechanical stress activates mechanosensitive pathways through ion channels (TRPV1, TRPV4, Piezo1, and Piezo2) expressed on synoviocytes. Both type A (macrophage-like) and type B (fibroblast-like) synoviocytes initiate intracellular signalling cascades, including NF-κB, MAPK, and PKC, that drive the production and release of pro-inflammatory cytokines (TNF-α, IL-1β, IL-6). These mediators recruit immune cells, polarise synoviocytes towards a catabolic phenotype, activate chondrocytes, and sustain synovial inflammation. In both cell types, pro-inflammatory signalling also enhances the production of proteolytic enzymes (e.g., MMPs), which degrade the ECM. This damage releases DAMPs that re-stimulate synoviocytes, amplifying inflammation and tissue breakdown in a reinforcing loop that perpetuates joint destruction. DAMPs: Damage-associated molecular patterns; ECM: extracellular matrix; IL-1β: Interleukin-1 beta; IL-6: Interleukin-6; MAPK: Mitogen-activated protein kinase; MMPs: Matrix metalloproteinases; NF-κB: Nuclear factor kappa-light-chain-enhancer of activated B cells; PKC: Protein kinase C; ROS: Reactive oxygen species; TNF-α: Tumour necrosis factor-alpha; TRPV1: Transient receptor potential vanilloid 1; TRPV4: Transient receptor potential vanilloid 4.

Synovial inflammation in degenerative joints was described nearly a century ago and is now recognised as both a consequence of tissue damage and a driver of OA progression [12]. It results from the convergence of systemic (metaflammation) and local (mechanoflammation) mechanisms that sustain a chronic inflammatory microenvironment that contribute to the structural damage of the OA knee (Fig. 1). Key pathogenic axes include excessive mechanical loading, activation of innate inflammatory pathways, and systemic disturbances linked to, for instance, obesity and metabolic syndrome (MetS) [5,13]. Obesity is particularly relevant, as it combines systemic metabolic dysfunction and chronic low-grade systemic inflammation with local biomechanical stress, illustrating how multiple pathways intersect to perpetuate synovitis.

#### 3.2.1 Metaflammation

Multiple studies have demonstrated that the metabolic alterations characteristic of MetS, including obesity and dyslipidaemia, are intimately involved in the initiation and persistence of synovial inflammation in OA. These alterations influence both weight-bearing joints, such as the knee, and non-weight-bearing joints, suggesting that mechanical load alone cannot fully account for OA pathogenesis [14,15]. Weight loss may slow effusion-synovitis, and localised fat reduction could play a role in managing inflammation associated with knee OA [16].

The mechanistic link appears to lie in the complex interplay between metabolic regulation and immune activation [17]. The chronic low-grade systemic inflammation observed in obesity is largely driven by the accumulation of visceral adipose tissue and the associated release of pro-inflammatory adipokines such as leptin, resistin, and visfatin, along with classical cytokines like tumour necrosis factor (TNF)-α and interleukin (IL)-6 [18].

Synovial adipocytes secrete adipokines into the synovial fluid, where they exert both catabolic and anabolic effects. The infrapatellar fat pad (IPFP) of OA patients produces higher levels of leptin and adiponectin than in healthy controls [19]. Adiponectin, part of the C1q-TNF superfamily, promotes production of prostaglandin (PG)E_2_, matrix metalloproteinase (MMP)-13, nitric oxide (NO), and pro-inflammatory cytokines (IL-6, monocyte chemoattractant protein (MCP)-1, C-C motif chemokine ligand (CCL)2 in OA chondrocytes [20], but may also inhibit IL-1β-induced MMP-13 and upregulate tissue inhibitor of metalloproteinases (TIMP)-2, suggesting context-dependent anti-inflammatory effects. A meta-analysis confirmed higher circulating adiponectin levels in OA, though without a clear correlation to disease severity [21].

Leptin, also secreted by the IPFP, is upregulated in OA cartilage and synovial fluid, where it enhances MMP-3, MMP-13, NO, and cytokine expression, contributing to a catabolic environment [22,23]. Its expression correlates with cartilage damage and may additionally promote chondrocyte proliferation and differentiation via anabolic pathways [23]. The dual role of adipokines reflects the IPFP's response to molecular cues from joint damage [19,20], amplifying local pro-inflammatory signalling. In animal models lacking functional leptin signalling or with lipodystrophy, cartilage is protected despite obesity or meniscal injury, while synovitis persists, indicating that disrupted adipose signalling uncouples mechanical stress from cartilage degeneration [24].

This pro-inflammatory environment is not confined to systemic circulation but extends locally to the synovial membrane, where it promotes the activation of innate immune responses. Macrophage infiltration, particularly polarised toward an M1-like pro-inflammatory phenotype, is commonly observed in the synovia of obese OA patients, contributing to synovial hyperplasia and cartilage catabolism [25]. The involvement of the innate immune system, both in adipose tissue and synovial tissue, supports a more aggressive, metabolically driven OA phenotype [17].

Recent experimental and clinical studies suggest that dyslipidaemia itself, independent of body mass, can exacerbate synovial inflammation. Animal models of hyperlipidaemia have shown increased synovial thickening and inflammatory cell infiltration, supporting the hypothesis that abnormal lipid metabolism can directly enhance joint inflammation [26] In this context, macrophages infiltrating the synovium may become laden with lipids, forming foam cells that contribute to synovial invasiveness and a more inflammatory phenotype of OA [27].

#### 3.2.2 Mechanoflammation

Although the upstream activators of mechanoflammation are still debated, it occurs when abnormal mechanical loads activate mechanoreceptors on the chondrocyte membrane. This activation triggers mechano-sensitive inflammatory signalling pathways, which are known to activate nuclear factor (NF)kB and inflammatory mitogen-activated protein (MAP) kinases, regulating aggrecanase activity and nerve growth factor production [28]. These pathways lead to the production of proteases, which in turn cause the degradation of the matrix. The released matrix fragments then induce synovial inflammation and pain [28,29].

*In vitro* models show that chondrocytes and synovial cells respond to mechanical stress by producing ECM-degrading enzymes and pro-inflammatory mediators [28,[30], [31], [32]] Clinical data support these findings: gait abnormalities and joint overload are associated with synovial inflammation, including perivascular oedema and increased vascularisation, both linked to more severe radiographic damage [33,34].

#### 3.2.3 Mechanotransduction of mechanical stress

Cells detect mechanical forces via the ECM through a process known as mechanotransduction, which triggers intracellular signalling and alters cell function [35]. In the osteoarthritic joint, several mechanosensory mechanisms contribute to inflammation and tissue remodelling. For instance, integrin activation on macrophages and synovial fibroblasts can trigger the release of matrix-sequestered growth factors, such as latent transforming growth factor (TGF)β, thereby influencing both immune and stromal cell function [36]. In parallel, a variety of mechanosensitive ion channels, such as transient receptor potential channels of the vanilloid subfamily (TRPV1, TRPV4) and, Piezo1, and Piezo2, are expressed in synovial fibroblasts and innate immune cells, where they regulate calcium flux and contribute to pro-inflammatory signalling [37]. Toll-like receptors (TLRs) also interact with these mechanoreceptors, facilitating ion entry and jointly modulating pro-inflammatory activation and macrophage polarisation [38].

Mechanosensory pathways converge with innate immune mechanisms via the detection of damage-associated molecular patterns (DAMPs). These endogenous molecules, released from injured tissue, activate TLRs on synovial cells and macrophages, triggering and sustaining the inflammatory response in OA [5,13]. Key DAMPs present in the osteoarthritic synovium include cartilage matrix fragments, oxidative stress derivatives, and molecules released during cell death, such as fibronectin fragments, HA, heparan sulphate, tenascin, fibrinogen, serum amyloid A, high-mobility group box 1 (HMGB1), and the alarmins S100A8/A9 [5,13,39].

Additionally, calcium-containing microcrystals, specifically basic calcium phosphate and calcium pyrophosphate, which are frequently found in the synovial fluid of OA patients, serve as potent DAMPs. These crystals activate the NOD-like receptor family, pyrin domain containing 3 NLRP3) inflammasome and TLRs in synovial cells, initiating signalling cascades involving protein kinase C, extracellular signal-regulated kinases 1 and 2 ERK1/2, part of the mitogen-activated protein kinase (MAPK) (ERK1/2 MAPKs), and nuclear factor kappa-light-chain-enhancer of activated B cells (NF-κB). The result is an increased production of pro-inflammatory mediators such as TNF-α, IL-6, and IL-1β, which contribute to chronic synovial inflammation and joint damage [5,13].

#### 3.2.4 Immune cells polarisation

The additional activation of innate immunity is a central mechanism in initiating and sustaining the inflammatory response in OA [5,13]. Recent technological advances, such as single-cell ribonucleic acid (RNA) sequencing, have uncovered a surprising heterogeneity of immune cells in osteoarthritic joints, particularly within the synovium and infrapatellar fat pad. In advanced stages of OA, organised immune cell aggregates, comprising B cells, T cells, and surrounding plasma cells, have been observed, suggesting localised immune activation [40]. The detection of circulating autoantibodies and shifts in memory T and B cell populations further point to a role for self-reactivity in OA pathogenesis. These immune alterations contribute to both local and systemic low-grade inflammation, which may differ across OA phenotypes and represent potential therapeutic targets. Similar mechanisms are observed in shoulder OA, where mechanical overload induces synovial fibrosis, neovascularisation, and macrophage polarisation, driven by molecular mechanosensors [41]. Both resident stromal cells and innate immune cells can detect and respond to biomechanical stress by initiating inflammatory cascades [42]. Likewise, the subchondral bone undergoes an uncoupled remodelling process, often marked by macrophage infiltration and osteoclast activity, which may precede visible cartilage degeneration [43,44].

#### 3.2.5 Structural degeneration from metaflammation and mechanoflammation

**Fig. 2:**
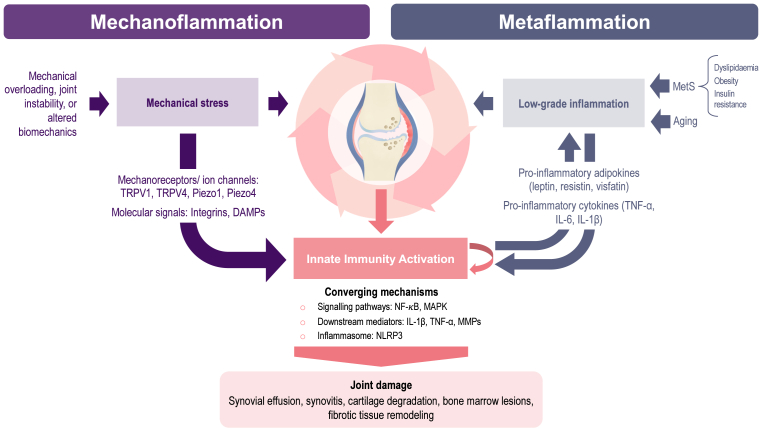
**Pathways linking mechanoflammation and metaflammation to joint damage – Footnote** This figure illustrates how mechanical stress (mechanoflammation, local) and metabolic dysfunction (metaflammation, systemic) act as parallel yet converging drivers of innate immune activation in knee OA. Although initiated by different upstream stimuli, mechanical overload versus metabolic dysregulation, both processes converge on shared intracellular signalling pathways, most notably NF-κB and MAPK. These pathways regulate the transcription of pro-inflammatory and catabolic mediators, including IL-1β, TNF-α, and MMPs. Activation of the NLRP3 inflammasome, particularly relevant in metaflammation, further sustains IL-1β production. Together, mechanoflammation and metaflammation establish a self-perpetuating cycle of cartilage degradation, synovial activation, and structural joint damage. DAMPs: Damage-associated molecular patterns; IL-1β: Interleukin-1 beta; IL-6: Interleukin-6; MAPK: Mitogen-activated protein kinase; MMPs: Matrix metalloproteinases; NF-κB: Nuclear factor kappa-light-chain-enhancer of activated B cells; NLRP3: NOD-, LRR- and pyrin domain-containing protein 3; OA: Osteoarthritis; TLRs: Toll-like receptors; TNF-α: Tumour necrosis factor-alpha; TRPV1: Transient receptor potential vanilloid 1; TRPV4: Transient receptor potential vanilloid 4.

Abnormalities in the suprapatellar fat pad (SPFP) and effusion-synovitis in the suprapatellar pouch are associated with knee pain, bone marrow lesions (BMLs), and biomarkers such as oligomeric matrix protein (COMP) and high-sensitivity C-reactive protein (hs-CRP), indicating their role in structural degeneration and inflammation [[45], [46], [47], [48]]. Knee pain is further linked to BMLs and effusion-synovitis, while elevated ghrelin levels correlate with increased symptoms, IPFP alterations, and matrix turnover markers, such as MMP-3, N-terminal telopeptide of type I collagen (NTX-I), and procollagen type III N-terminal propeptide (PIIINP) [49]. Together, these local tissue markers (COMP, MMP-3, NTX-I, PIIINP) and systemic metabolic–inflammatory factors (ghrelin, hs-CRP) suggest a dual contribution of local and systemic processes to knee OA pathophysiology [[30], [31], [32]] (Fig. 2). Within the synovium, fibroblast-like synoviocytes and macrophages integrate signals from both domains, amplifying inflammatory cascades.

The relationship between synovitis and structural change, however, is complex. Some evidence suggests that effusion-synovitis can drive cartilage defects, particularly in early OA [19,50,51], whereas other longitudinal studies indicate that synovial inflammation more often reflects ongoing structural damage in established disease [52,53]. Indeed, cartilage and subchondral bone abnormalities have been shown to predict changes in effusion-synovitis, while the reverse is not consistently observed [54].

## 4 Biochemical, imaging and clinical markers of knee OA

### 4.1 Biochemical markers

Increased serum levels of MMP-3 have been associated with synovitis and effusion [55] while serum levels of ghrelin have been linked to increased knee symptoms, such as worse pain, stiffness and dysfunction [54]. Magnetic resonance imaging (MRI)-detected alterations in IPFP signal intensity, along with elevated serum levels of MMP-3 and MMP-13, NTX-I, and PIIINP may also reflect inflammation and tissue catabolism, potentially correlating with pain and disease progression, active cartilage degradation, increased bone resorption and subchondral bone changes, tissue remodelling and fibrosis in the joint structures [19,51]. Cluster of differentiation (CD)14, CD163, type II collagen C-propeptide, TNF-stimulated gene-6, IL-6, TNF-α, and aggrecan release are associated with disease progression [56]. There is a positive correlation between OA severity and the levels of vascular endothelial growth factor (VEGF), chemokine (C-C motif) ligand 2 (CCL2), leptin, and tissue inhibitor of metalloproteinase-1 (TIMP-1) [56]. Levels of chondroitin sulphate (C4S), tenascin-C, and aggrecan may predict the efficacy of HA injections, while white blood cell counts can predict the response to steroid injections [56].

Together, these findings highlight a broad range of biomarkers, such as serum MMP-3, ghrelin, MMP-13, NTX-I, and PIIINP, correlate with MRI-detected synovitis, effusion, pain and functional impairment, and treatment outcomes, highlighting their potential role in monitoring disease activity and therapeutic response.

### 4.2 Imaging markers

Imaging plays a crucial role in assessing synovitis in OA, with MRI and ultrasound being the most commonly used modalities [57]. MRI offers exceptional visualisation of joint structures involved in degeneration. Three key outcomes to consider are the prediction of radiographic knee OA incidence, OA progression, and knee arthroplasty risk [58]. The Joint Narrowing Score (JNS), Kellgren–Lawrence (KL) scores, and trabecular bone texture features are potential imaging biomarkers for these outcomes [58].

There is growing interest in quantitative MRI techniques for detecting early biochemical and microarchitectural changes before structural damage in OA [59]. T2 relaxation time mapping and T1ρ imaging are validated for early cartilage damage detection. Delayed gadolinium-enhanced MRI assesses cartilage integrity with contrast agents, while gadolinium-free methods like sodium imaging, glycosaminoglycan chemical exchange saturation transfer, and diffusion MRI also show promise. Advances in MRI hardware and image analysis tools, such as segmentation, improve speed and repeatability, making these techniques more suitable for clinical use [58,59].

In association with imaging, artificial intelligence (AI) is increasingly used in radiology to assist in detecting patterns and correlations in knee OA that may be useful in diagnosis, grading, predicting the need for arthroplasty, and improving surgical accuracy. AI offers potential for analysing knee pathologies and reducing radiologists' workload, although supervision is typically necessary [58,59].

Brain imaging is an emerging field for studying the relationship between brain changes and pain in knee OA. Reported findings include structural changes like cortical thinning and functional changes such as altered cerebral blood flow, disrupted functional connectivity, and abnormalities in the pain connectome, particularly in the right anterior insula [60]. However, a detailed examination of the relationship between synovitis, progression and pain is beyond the scope of this article. Further research is needed to understand brain abnormalities related to chronic pain and knee OA, and the potential benefits of treatments extending beyond the knee [61].

### 4.3 Knee pain as a clinical marker

Within the group experiencing knee pain, distinct pain trajectories have been identified that correlate with self-reported function and are consistent across studies. These OA pain phenotypes include low-fluctuating, mild-increasing, moderate-treatment-sensitive, and severe-treatment-insensitive pain, with the moderate and severe pain groups being minorities among those with painful radiographic knee OA [62].

The risk of individuals transitioning from non-painful to painful radiographic knee OA, or vice versa, remains largely unexplored. The shift from painful to non-painful radiographic OA may reflect disease resolution. It could represent successful remodelling in OA, possibly driven by fibrotic processes that influence the expansion of the synovial ECM and lead to reduced vascularisation and pain.

Notably, a substantial proportion of individuals with radiographic knee OA remain asymptomatic, even in the presence of advanced structural changes [49,62]. In a large comparative study using data from the Hallym Aging Study (n = 504), the Korean National Health and Nutrition Examination Survey (n = 10,152) and the U.S.-based Osteoarthritis Initiative (n = 4796), between 24 % and 36 % of individuals with KL grade 3 or 4 did not report knee pain [63]. Among those with early radiographic changes (KL grades 1–2), less than 10 % reported knee pain. The likelihood of pain tends to increase with higher KL grades and greater joint space narrowing, particularly among individuals with obesity [63,64].

The volume of vascular-rich synovitis may explain the mismatch between radiographic changes and pain, particularly when measured relative to bone size in younger people with mild to moderate OA. Perimeniscal and infrapatellar synovitis are closely linked to pain, while higher levels of fibrosis markers are negatively associated with pain [49].

### 4.4 From clinical phenotypes to molecular endotypes in knee OA

OA is increasingly recognised as a heterogeneous disease with distinct clinical phenotypes and molecular endotypes [52,65]. Clinical OA phenotypes include subsets such as age-related, obesity-related, post-traumatic and metabolic subtypes, each presenting with unique symptoms and progression patterns [53].

For instance, age-related OA typically presents with gradual cartilage thinning, reduced regenerative capacity, and often lower levels of synovial inflammation. Post-traumatic OA usually develops early and progresses rapidly after joint injuries, whereas metabolic OA, linked to obesity, insulin resistance, and dyslipidaemia, is characterised by systemic inflammation, accelerated structural damage and altered joint metabolism [66,67].

In contrast to clinical phenotypes, molecular endotypes refer to the underlying biological mechanisms driving these phenotypes, such as inflammation, cartilage degradation, or metabolic dysregulation. These endotypes reflect a molecular taxonomy of the disease [4] defined by distinct molecular entities and cellular pathways involved in the pathogenesis of knee OA. For example, some endotypes are driven by pathways related to repair and inflammation, others by fibrosis and low repair [52].

One well-established example is the inflammatory endotype, characterised by high synovial levels of IL-1β, IL-6, TNF-α, and markers of macrophage activation. This form of OA involves low-grade synovial inflammation that contributes to joint damage, heightened nociception, and disease progression [68]. The structural damage endotype is defined by elevated markers of cartilage and bone degradation, such as MMPs and collagen fragments. A recent study showed that approximately 59 % of patients consistently fell into this category over time [69]. The low-turnover or fibrotic endotype is marked by poor tissue repair, elevated TGF-β signalling, and excessive ECM deposition, commonly observed in older adults with reduced regenerative capacity [52]. Meanwhile, obesity-driven endotypes feature altered synovial transcriptomes influenced by adipokines, insulin resistance, and lipid metabolism, affecting both load-bearing and non-load-bearing joints [70].

Importantly, molecular endotypes appear to be relatively stable over time. In a recent biomarker-based study (APPROACH/SMC), more than 50 % of participants maintained the same molecular profile, whether inflammatory, structural, or fibrotic, over two years, suggesting these endotypes are not transient and could help guide personalised therapeutic approaches [69].

### 4.5 Therapeutic approaches in knee OA

Non-pharmacological measures, education, self-management, exercise, weight loss, and walking aids, are widely recommended as first-line treatments [3]. Pharmacological strategies remain largely symptomatic [71], underlining the need for more effective options, including advanced analgesics and disease-modifying OA drugs (DMOADs).

Despite decades of research, no DMOAD has gained regulatory approval. Approved pharmacological treatments remain limited to analgesics, oral and topical NSAIDs, and symptomatic slow-acting drugs [[72], [73], [74]]. Intra-articular (IA) therapies, corticosteroids and viscosupplements are typically used when conservative measures fail [74,75]. with surgery as a last resort [3,75].

Several promising therapies have now entered phase 2 and 3 trials [76]. The therapeutic focus is shifting from systemic agents to targeted IA delivery based on OA endotypes, improving joint bioavailability and reducing systemic toxicity [77,78].

### 4.6 Therapies in clinical use

#### 4.6.1 Oral drugs targeting mechanoflammation

Oral drugs for OA synovitis in clinical use include NSAIDs, paracetamol, colchicine, and diacerein (Table 1). NSAIDs are the first-line treatment for knee OA due to their efficacy in reducing pain, inflammation, and effusion size [79,80]. However, they pose risks for patients with gastrointestinal and cardiovascular conditions, and long-term use may worsen synovitis and cartilage degradation [74,81]. Paracetamol may reduce synovial effusion and tissue volume in OA, though its anti-inflammatory effects are less documented compared to NSAIDs [74]. The combined use of oral paracetamol and topical NSAIDs is widely accepted among healthcare professionals, despite the limited evidence supporting the efficacy of this combination [82]. Colchicine, typically used for gout, has shown mixed results in OA, with some studies suggesting reduced joint replacement incidence, though its efficacy in symptom management remains uncertain [83]. Diacerein, which inhibits IL-1, is non-inferior to celecoxib in reducing pain and improving function, but it is associated with more adverse events, particularly diarrhoea [84].

**Table 1:** Investigational oral and IA pharmacological therapies for knee OA synovitis.

Therapy	Mechanism of action	Effect on synovitis	Clinical effect	Adverse events/limitations
Oral				
NSAIDs	Inhibit COX enzymes, reducing prostaglandin production.	Reduce joint pain and effusion size; lower markers of cartilage and synovial metabolism.	Effective in alleviating pain and improving function.	Risk of gastrointestinal and cardiovascular undesirable effects.
				Long-term use may worsen synovitis and cartilage degeneration.
Paracetamol	Unclear, potentially anti-inflammatory.	Reduces synovial effusion and tissue volumes.	Reduces synovial effusion and tissue volumes in knee OA.	Minimal.
			Generally used in combination with other treatments due to limited efficacy as monotherapy.	
Colchicine	Inhibits microtubule assembly and disrupts inflammasome activation.	Potential reduction in synovitis.	Mixed results on OA symptom improvement.	Risk of gastrointestinal undesirable effects.
Diacerein	Inhibits IL-1 pro-inflammatory effects.	Not specifically studied for OA synovitis.	Reduces pain, improves function (similar to celecoxib).	Diarrhoea (increased incidence of undesirable effects compared to celecoxib).
IA				
Corticosteroids	Reduce synovial inflammation by inhibiting proinflammatory cytokines (IL-1, IL-6, TNF-α) and decreasing COX-2 expression.	Significantly reduces synovial tissue volume and synovial infiltration in OA joints.	Effective in the short-term (2–4 weeks), especially in severe pain cases.	Regular injections not recommended due to cartilage volume loss over 2 years.
	Inhibits T-cell generation, proliferation, and activation.	Quantifiable synovial tissue reduction and rebound in individuals experiencing pain relapse.	No significant pain relief compared to placebo after 3 months.	
Hyaluronic Acid	Provides joint lubrication and chondroprotection.	Improves synovitis, reduces synovial membrane's inflammatory score, and decreases lining synoviocytes, blood vessel permeability, synoviocyte hypertrophy, vacuolized cells, necrotic areas, and macrophages.	Effective and safe, outperforming oral NSAIDs and IA corticosteroids in pain reduction and functionality. Repeated injections maintain these benefits without increased risks.	Mixed evidence. Small improvement in pain and function compared to placebo.
	Offers anti-inflammatory, mechanical, and analgesic benefits.			
	Enhances proteoglycan and glycosaminoglycan synthesis.			
Platelet-Rich Plasma	Provides high concentrations of growth factors (tissue growth factor, platelet-derived growth factors).	Reduces NF-kb signalling, reverses inhibition of type II collagen and aggrecan gene expression.	No consistent evidence of benefit in improving OA symptoms.	Limited evidence of long-term benefit.
	Enhances NF-kb inhibitor expression, reducing downstream inflammatory cytokine activation.		Well-designed trials do not show significant efficacy.	

#### 4.6.2 Intra-articular injections targeting mechanoflammation

Intra-articular (IA) injections are commonly used to manage OA synovitis, with corticosteroids, HA, and platelet-rich plasma (PRP) being the main options (Table 1). Corticosteroid injections reduce synovial inflammation and pain in the short term (2–4 weeks), especially in patients with severe pain or high synovial inflammation [85]. However, their long-term efficacy is limited, and regular use may lead to cartilage volume loss [86,87].

HA is the most commonly used biomaterial for viscosupplementation, primarily providing joint lubrication and chondroprotection [74]. HA injections provide lubrication and anti-inflammatory effects, with improvements in pain and function lasting up to six months. Repeated injections maintain these benefits without increased risks [88]. The benefits of HA are enhanced by high molecular weight formulations. However, evidence is variable, and some studies suggest only small improvements compared to placebo [[89], [90], [91]]. Guidelines remain inconclusive due to variable evidence [[92], [93], [94], [95]]. PRP, though promising due to its growth factor content and anti-inflammatory effects, has not shown consistent benefits in clinical trials for knee OA, particularly in older populations [[96], [97], [98], [99]].

#### 4.6.3 Therapies on investigation

The pro-inflammatory cytokines TNF-α and IL-1β are key drivers of catabolic signalling in affected joints, with IL-1β being a product of inflammasome activation [100]. Inflammasomes are inflammatory multiprotein complexes that propagate inflammation in various autoimmune and autoinflammatory conditions. They do this by inducing cell death and triggering the release of inflammatory cytokines and damage-associated molecular patterns [101,102], a process that, in the context of OA, is exacerbated by mechanoflammation [29].

#### 4.6.4 Intra-articular therapies targeting mechanoflammation

New therapies targeting local synovial inflammation include several innovative approaches (Table 2). One emerging therapy targeting mechano-inflammation is the IA injection of self-lubricating, lipid-incorporating hydrogels [103], with a new randomised controlled trial (including a placebo arm) currently nearing completion. The IA injection of carboxymethyl-chitosan, a novel biomaterial implant composed of 2.0 % non-crosslinked chitosan derivatives of non-animal origin obtained through proprietary chemistry from the edible white mushroom *Agaricus Bisporus*, showed significantly higher free radical scavenging capacity than HA formulations in the *in vitro* Trolox Equivalent Antioxidant Capacity assay [104,105].

**Table 2:** Investigational intra-articular therapies for knee OA synovitis.

Therapy	Description	Key findings
Carboxymethyl-chitosan	Intra-articular injection of 2.0 % non-crosslinked chitosan derivatives from edible white mushroom *Agaricus bisporus*.	Higher free radical scavenging capacity than HA, suggesting a more effective reduction in oxidative stress within the joint. This can potentially lead to reduced inflammation, slowed cartilage degradation, improved pain management, and enhanced joint function, reducing the progression of knee OA compared to HA alone.
2.5 % polyacrylamide hydrogel injection, iPAAG	2.5 % polyacrylamide hydrogel injection shown to reduce pain and improve function in knee OA patients.	Effective for at least 4 years, it integrates into the synovial membrane. Its clinical effects are derived from its chemical and biological inertness, meaning it does not actively direct any action. However, it may have an anti-inflammatory potential impact by disrupting interactions between inflammatory cells.
Nano-PAZII	Nanoparticle formulation of the VEGFR1/2 inhibitor pazopanib (dual action).	Sensory nerve ingrowth and neuronal plasticity are suppressed by VEGFR1 inhibition; cartilage integrity is preserved by VEGFR2 blockade. Prolonged pain relief; reduced cartilage damage; no notable side effects (pre-clinical).
Cryoneurolysis (Iovera° system)	Cryoneurolysis with the Iovera° system is a minimally invasive procedure that provides long-lasting pain relief by temporarily freezing peripheral nerves, disrupting their ability to transmit pain signals.	Effective for up to 150 days, significant pain reduction and improved symptoms, well-tolerated.

HA: Hyaluronic acid; iPAAG: Injectable polyacrylamide hydrogel; KL: Kellgren–Lawrence (radiographic grading system for osteoarthritis); OA: Osteoarthritis; PAZII: Pazopanib (VEGFR1/2 inhibitor) nanoparticle formulation; VEGFR1: Vascular Endothelial Growth Factor Receptor 1; VEGFR2: Vascular Endothelial Growth Factor Receptor 2.

A recent promising development is the 2.5 % polyacrylamide hydrogel injection (iPAAG). This biocompatible, nonabsorbable, and non-biodegradable cross-linked polyacrylamide hydrogel was initially used in veterinary applications for horses, and has recently received marketing authorisation in Europe and Canada [106]. Clinical studies over the past decade have demonstrated its efficacy in reducing pain and improving function in patients with symptomatic knee OA. Significant and clinically meaningful improvements in pain and function have been observed in patients with moderate to severe knee OA at 12 weeks, sustained through 52 weeks [[107], [108], [109]]. Results indicate a significant reduction in the WOMAC pain subscale (−12·1 units) and patient global assessment, with no adverse events related to treatment. Recent data show that a single 6 ml iPAAG injection is well-tolerated and effective in reducing pain for at least 4 years following treatment [110,111].

Unlike HA, which functions solely as a viscosupplement, iPAAG integrates into the synovial membrane of the inner joint capsule, thickening the synovial membrane. Its non-absorbable, non-biodegradable, and non-migratory properties provide durable cushioning to the inner capsular tissue, thereby reducing pain, decreasing joint stiffness, and improving the function of OA-affected knees. Unlike other intra-articular injections, iPAAG physically becomes an integrated part of the synovial tissue within the joint capsule, allowing it to exert its effects for a substantially longer duration than older OA treatments. While its clinical effects primarily stem from its chemical and biological inertness, meaning it does not actively direct any biological processes, it may have potential anti-inflammatory effects by disrupting interactions between inflammatory cells [112]. Ongoing research is focused on better understanding its exact mechanism of action.

Among the mediators not yet discussed in this manuscript are the vascular endothelial growth factor receptors (VEGFRs), with VEGFR1 implicated in pain and VEGFR2 in cartilage degeneration [113]. To target both, Nano-PAZII, a nanoparticle formulation of the VEGFR1/2 inhibitor pazopanib, was developed. In two OA rodent models (surgical and chemical induction), a single IA injection of Nano-PAZII provided prolonged pain relief and reduced cartilage damage without notable side effects [114]. It is hypothesised that VEGFR1 inhibition suppressed sensory nerve ingrowth and neuronal plasticity, while VEGFR2 blockade preserved cartilage integrity, supporting Nano-PAZII as a dual-action, DMOAD [115].

Small extracellular vesicles (sEVs) may also influence the OA joint microenvironment by delivering regulatory RNAs to chondrocytes. sEVs from OA synovial fibroblasts altered gene expression, reduced viability, and increased inflammatory cytokine production in chondrocytes, as well as enhanced extracellular matrix degradation in cartilage explants, highlighting their potential role in OA pathogenesis and as therapeutic targets [116].

Cryoneurolysis of the infrapatellar branch of the saphenous nerve resulted in significantly decreased knee pain and improved symptoms compared to sham treatment for up to 150 days and was well tolerated in a randomized, double-blind, sham-controlled, multicentre trial with a 6-month follow-up in patients with mild-to-moderate knee OA [117]. The Iovera® system, used to destroy tissue during surgical procedures by applying freezing cold, can also produce lesions in peripheral nervous tissue by applying cold to block pain [118]. It is also indicated for the relief of pain and symptoms associated with knee OA for up to 90 days [117,118].

#### 4.6.5 Oral and subcutaneous therapies targeting metaflammation

There are oral and subcutaneous therapies targeting metaflammation that are currently in trial (Table 3). Oral DFV890 is a NLRP3 inflammasome inhibitor currently in phase 2 trials for knee OA [119,120]. IL-1β inhibitors, such as anakinra and canakinumab, have shown efficacy in preventing cartilage breakdown in animal models, but human trials have not demonstrated significant pain relief. Exploratory studies suggest that IL-1 inhibition may reduce joint replacement rates, so further research is needed [121]. TNF-α, produced by activated synoviocytes, mononuclear cells, and articular cartilage, is a key cytokine involved in joint matrix destruction. Together with IL-1β, it activates inflammatory pathways leading to NF-kb activation, which regulates chemokines, cytokines, adhesion molecules, and matrix-degrading enzymes. TNF-α inhibitors like infliximab and adalimumab have shown potential in reducing joint damage in hand OA, though results are mixed in terms of pain reduction [122].

**Table 3:** Investigational pharmacological therapies targeting metaflammation in knee OA synovitis.

Therapeutic	Mechanism of action	Effect on synovitis	Clinical effects/adverse events/limitations
DFV890	Oral NLRP3 inhibitor targeting the inflammasome pathway.	Currently under Phase 2 study for knee OA patients.	Researched in patients with COVID-19 pneumonia. No results available on knee OA at the time of elaborating this publication.
Methotrexate	Folic acid antagonist with anti-proliferative and anti-inflammatory effects.	PROMOTE a randomised controlled phase 3 trial of treatment effectiveness.	High levels of cytokines and immune cell infiltration in osteoarthritis joints support the use of methotrexate for symptom relief. Well-tolerated for long-term use in most patients. Significant OA pain reduction at six months.
	Anti-proliferative action: inhibits purine and pyrimidine synthesis by antagonising folic acid.		
	Anti-inflammatory action: at low doses (15–25 mg/week), increases adenosine release, reducing inflammation.		
	Reduces pro-inflammatory cytokines, such as TNFα and IL-6, lowering inflammation and synovitis.		
Anakinra	IL-1β inhibitor, reducing inflammation and extracellular matrix remodelling	Prevent cartilage breakdown in animal models.	Mixed results.
			No significant pain reduction in human trials. Some trials show no improvement in OA symptoms compared to placebo
Canakinumab	IL-1β inhibitor.	Lower rates of hip and knee arthroplasty in patients with elevated CRP.	Direct evidence is needed for OA management.
Lutikizumab	Anti-IL-1α/β dual variable domain immunoglobulin.	Improves WOMAC pain score at week 16, with no significant changes beyond.	No significant changes in MRI-assessed synovitis or other endpoints.
Infliximab	Chimeric monoclonal antibody against TNF-α.	Potential effect on disease progression in erosive hand OA.	Reduction in anatomic lesion scores; mixed results in various studies.
Adalimumab	Fully human monoclonal antibody against TNF-α.	Mixed results in hand OA treatment	Some trials show no improvement in pain; others show a reduction in new erosions.
Pentosan Polysulphate Sodium	Targets inflammation by inhibiting ADAMTS4, ADAMTS5, and NF-κB.	Reduces pain by inhibiting NGF expression in osteocytes.	Improved pain and function scores compared to placebo.
		Significant reductions in synovial fluid biomarkers.	Potential candidate for OA treatment.

ADAMTS4: A Disintegrin and Metalloproteinase with Thrombospondin motifs 4; ADAMTS5: A Disintegrin and Metalloproteinase with Thrombospondin motifs 5; CRP: C-reactive protein; DFV890: Oral NLRP3 inflammasome inhibitor; IL-1α: Interleukin-1 alpha; IL-1β: Interleukin-1 beta; IL-6: Interleukin-6; NGF: Nerve Growth Factor; NF-κB: Nuclear Factor kappa-light-chain-enhancer of activated B cells; NLRP3: NOD-like receptor family, pyrin domain containing 3; OA: Osteoarthritis; PROMOTE: Pain Reduction with Oral Methotrexate in Knee Osteoarthritis (phase 3 clinical trial); TNF-α: Tumour Necrosis Factor alpha; WOMAC: Western Ontario and McMaster Universities Osteoarthritis Index.

Pentosan polysulphate sodium, delivered subcutaneously, has demonstrated significant reductions in pain and synovial fluid biomarkers in knee OA patients, suggesting it may be a promising DMOAD [123].

Methotrexate is a folic acid antagonist with both anti-proliferative and anti-inflammatory effects. It inhibits purine and pyrimidine synthesis to exert its anti-proliferative action, while at lower doses (15–25 mg/week), it increases adenosine release, reducing inflammation by lowering pro-inflammatory cytokines such as TNFα and IL-6 [124]. The PROMOTE phase 3 trial demonstrated significant OA pain reduction at six months, with methotrexate being well-tolerated for long-term use [125]. High levels of cytokines and immune cell infiltration in OA joints provide further rationale for its use in symptom relief [124,125].

Low-dose radiotherapy is commonly used in some European countries for OA pain relief. The primary effect of low dose ionizing radiation, leading to symptom improvement, is through the reduction of synovial effusion and synovial cell proliferation [126,127].

#### 4.6.6 Multitargeting therapy

Multitargeting therapies for knee OA focus on addressing multiple underlying mechanisms of the disease to provide more effective treatment [52,65,69] by reducing cartilage degeneration, inhibiting chondrocyte hypertrophy, and controlling inflammation that worsens the disease (Table 4). By simultaneously addressing both structural damage and the inflammatory response, a multitargeting approach has the potential to slow disease progression, alleviate pain, and improve joint function. It would also allow for more personalised treatment, catering to the different endotypes or subtypes of OA, offering a more effective approach to managing the condition from multiple angles.

**Table 4:** Therapies for knee OA synovitis by clinical use, investigational status, endotype, and phenotype.

Endotype	Clinical phenotype	Therapy or method	Target in synovitis	Clinical objective
Therapies in clinical use				
Inflammatory	Pain, swelling, effusion	NSAIDs (oral/topical)	COX inhibition, reduces prostaglandin-mediated inflammation	Pain and inflammation control
Non-inflammatory	Mild pain, limited swelling	Paracetamol	Unclear anti-inflammatory action; mild symptom relief	Pain control
Metabolic/inflammatory	Fluctuating pain, possible crystal arthropathy	Colchicine	Inhibits microtubule assembly, reduces leukocyte activity	Reduce flares, joint replacement risk
Inflammatory	Pain, mild effusion	Diacerein	IL-1 inhibition	Pain relief, function improvement
Inflammatory	Acute synovitis, severe pain	IA corticosteroids	Suppress synovial inflammation	Short-term relief
Mechanical/inflammatory	Moderate OA, pain with activity	HA injections	Joint lubrication, mild anti-inflammatory effects	Improve function, reduce pain
Inflammatory	Younger patients, early OA	PRP injections	Growth factors, anti-inflammatory cytokines	Modest symptom relief, tissue modulation
Inflammatory	Persistent effusion, systemic features	Methotrexate	Adenosine-mediated cytokine suppression	Pain reduction, inflammation control
Inflammatory	Severe synovitis, treatment-resistant	Low-dose radiotherapy	Reduces synovial proliferation	Symptom improvement
Investigational therapies				
Mechanical/inflammatory	Moderate-severe OA, structural instability	iPAAG hydrogel	Synovial integration, cushioning	Long-term pain and stiffness reduction
Metabolic/inflammatory	Obese/metabolically active OA phenotype	DFV890	Inflammasome inhibition	Modulate systemic/local inflammation
Inflammatory	High synovitis, early cartilage loss	Anakinra, Canakinumab	IL-1β blockade	Delay structural progression
Inflammatory	Multi-joint symptoms, RA overlap	Infliximab, Adalimumab	TNF-α inhibition	Reduce inflammation, limit joint damage
Inflammatory	High pain, elevated synovial biomarkers	Pentosan polysulfate	Cartilage metabolism modulation	Pain relief, biomarker reduction
Oxidative/inflammatory	Pain, oxidative stress markers	IA chitosan injection	Free radical scavenging, antioxidant	Pain control, inflammation modulation
Vascular/inflammatory	Effusion, synovitis with vascular hyperplasia	GAE	Reduces synovial blood flow	Short-term pain/function improvement
Neurogenic	Localised pain, refractory symptoms	Cryoneurolysis	Disrupts nociceptive signalling	Short- to medium-term pain relief

IA: intraarticular; HA: hyaluronic acid; iPAAG: injectable polyacrylamide gel; OA: osteoarthritis; PRP: platelet-rich plasma; RA: rheumatoid arthritis.

#### 4.6.7 Gene therapies

According to the US Food and Drug Administration (FDA), human gene therapy aims to modify or manipulate gene expression or alter the biological properties of living cells for therapeutic use [128]. This technique can treat or cure diseases by replacing a disease-causing gene with a healthy copy, inactivating a malfunctioning gene, or introducing a new or modified gene to help treat a disease. In contrast to conventional treatments gene therapy enables the prolonged expression of therapeutic proteins precisely at targeted sites [129].

Several types of gene therapy products that offer promising avenues for treating knee OA, both directly and indirectly, through diverse mechanisms and therapeutic strategies [128]. The synovium is a central focus of several gene- and cell-based therapies serving as a site of delivery, a primary source of inflammation, or a key modulator of broader joint pathology. In these approaches, synovitis is not just a consequence of OA but also considered a therapeutic target in itself (Table 5).

**Table 5:** Investigational gene therapies for knee OA synovitis.

Approach	Therapy/Method	Mechanism of action	Target in synovitis	Clinical objective
**Direct targeting of synovial inflammation**	**Allocetra**	Reprograms macrophages to resolve synovitis.	Synovial macrophages and immune response.	Reduce synovial inflammation and improve joint function.
	**PLX-PAD**	Placental stromal cells with immunomodulatory properties, delivered intra-articularly.	Chronic synovial inflammation and cartilage degradation.	Alleviate inflammation and promote cartilage preservation.
	**PCRX-201**	Inflammation-responsive gene expression of IL-1Ra via adenoviral vector.	Inflamed synovial tissue.	Produce local anti-inflammatory effects.
**Indirect modulation via immune/joint environment**	**Mesenchymal stem cells**	Immunomodulation, reduced IL-10, improved synovial markers	Immune and inflammatory pathways in synovium.	Improve pain/function, reduce synovial inflammation
	**TissueGene-C**	Chondrocyte-based TGF-β1 overexpression.	Indirectly alters cytokine milieu and joint homeostasis.	Promote regeneration, potentially reduce low-grade synovitis.
	**RMD1101**	FGF-18-based regenerative mechanism.	May modulate synovial cytokine activity.	Support cartilage repair and influence synovial balance.
	**Stromal Vascular Fraction**	Autologous adipose-derived cells, including macrophages and pericytes, reduce immune activation.	Synovial immune cell environment.	Reduce inflammation, improve pain and mobility.
**Targeted delivery to synovial tissue**	**Viral vectors**	Efficient gene delivery (e.g. adenovirus) to synovial cells.	Synovial tissue as delivery site and therapeutic target.	Prolonged local therapeutic expression.
	**Non-viral methods**	Electroporation and chemical delivery enhance gene uptake in synovial cells.	Synovial membrane as a direct gene therapy target.	Improve the specificity and efficacy of intra-articular delivery.

FGF-18: Fibroblast Growth Factor 18; IL-1Ra: Interleukin-1 receptor antagonist; IL-10: Interleukin-10; PLX-PAD: Placental expanded–placental adherent stromal cells; PCRX-201: Inflammation-responsive adenoviral gene therapy encoding IL-1Ra; RMD1101: Recombinant Fibroblast Growth Factor 18; TGF-β1: Transforming Growth Factor beta 1.

#### 4.6.8 Cell-based gene therapies

Cell-based gene therapies hold significant promise in the realm of OA, representing a paradigm shift by targeting underlying molecular mechanisms implicated in OA pathophysiology. These therapies involve delivering genetic material encoding therapeutic proteins (typically growth factors, cytokines, or anti-inflammatory molecules) into joint tissues to modulate cellular activities and promote cartilage repair and regeneration. To date, several cell types and gene delivery systems have been explored in preclinical and clinical studies for OA treatment [130].

#### 4.6.9 TissueGene-C

TissueGene-C is a novel gene and cell therapy consisting of human allogeneic chondrocytes and irradiated GP2-293 cells overexpressing TGF-b1 [131]. TGF- b plays a role in cellular differentiation, the synthesis of ECM, and chondrogenesis. TGF- b signalling pathways are believed to be involved in early cartilage development and the maintenance of cartilage over time. Results from two small phase II trials and one phase III trial demonstrated statistically significant improvements in pain and function in patients, with trends of structural change [[132], [133], [134]].

#### 4.6.10 RMD1101

RMD1101 is a novel gene therapy being investigated as a single-injection treatment based on the regenerative mechanism of the fibroblast growth factor 18 protein hypothesis [135]. Stromal vascular fraction involves autologous adipose-derived stem cells mixed with other cells like macrophages, endothelial cells, and pericytes, reducing immune reactions.

#### 4.6.11 Mesenchymal stem cells

The utility of mesenchymal stem cells (MSCs) lies in their capacity for self-renewal, plasticity, and differentiation into different cell types like bone cells or cartilage [136,137]. They can also regulate immune response, reduce inflammation and apoptosis, and stimulate angiogenesis. Several studies have shown that IA injection of MSCs (mainly from autologous origin) is safe and efficacious [95,136,137]. Low doses led to significant improvement in pain and function, with safety confirmed over a six-month follow-up. However, their effect may not be sufficient for complete regeneration and recover [136]. For instance, patient-perceived clinical improvements and decreased synovial inflammation have been reported following a single IA injection of bone-marrow-derived MSCs [138]. Likewise, a significant reduction in IL-10 concentration in the synovial fluid, accompanied by decreased symptoms 12 months has been shown after IA injection [139]. A phase I clinical trial examined IA application of adipose-derived stromal cells in patients with knee OA [140,141]. IA injections with stromal vascular fraction have clinically decreased pain and increased joint mobility [142].

#### 4.6.12 Allocetra

Allocetra is an experimental cell therapy originally designed for immune modulation in conditions such as sepsis and organ transplant rejection. The therapy takes advantage of an apoptotic cell-based intervention that enhances the innate ability to exert systemic immunomodulation [[143], [144], [145]]. Its mechanism involves reprogramming macrophages to resolve synovitis and by modulating the synovial immune response, Allocetra potentially has the capacity to reduce this inflammation, possibly slowing the progression of the disease and improving joint function. While promising, Allocetra's application in OA is still in early developmental stages, requiring further clinical trials to confirm its safety and efficacy.

#### 4.6.13 PLX-PAD

Pluri's PLacental-eXpanded (PLX-PAD) stromal cells represent a novel and advanced regenerative medicine cell therapy focused on the use of mesenchymal-like adherent stromal cells (ASCs) isolated from the placenta to treat critical limb ischemia [146]. PLX-PAD cells have also been investigated in musculoskeletal trials investigating improved mobilisation after hip fracture [147], sarcopenia and tendon injuries [148]. In this context, the therapeutic potential of PLX-PAD can be leveraged to promote tissue repair, reduce inflammation, and stimulate cartilage regeneration in OA. This justification is that these cells have immunomodulatory properties, which help alleviate chronic inflammation that contributes to cartilage degradation in OA. Pluri's PLX-PAD therapy is designed for intra-articular administration to the affected joint. It has demonstrated potential in preclinical and early-stage clinical studies to enhance joint function and alleviate pain. However, further trials are needed to validate its long-term efficacy in targeting synovitis.

#### 4.6.14 Vector-based gene therapies

Both viral and non-viral vectors can deliver genes to target cells [149]. Viral vectors like retroviruses, adenoviruses, and adeno-associated viruses are commonly used [129]. Adenovirus is advantageous for delivery efficiency in synovial tissue, while adeno-associated viruses are suitable for both *in vitro* and *in vivo* DNA therapy in the synovium. Helper-dependent adenoviruses seem more efficient for cartilage [129].

PCRX-201 is an intra-articular high-capacity adenoviral gene therapy vector that codes for the expression of IL-1Ra [150]. Its design includes an inducible promoter that activates only in the presence of inflammation signals, transforming joint cells into factories that produce sustained therapeutic levels of IL-1Ra.

Non-viral vectors deliver RNA or DNA physically or chemically through the cell membrane into the target cell [151]. Electroporation, a safe and efficient physical method that applies a high-voltage impulse to the cell membrane, can stimulate cellular uptake in synovial cells [151]. XT-150, a non-viral pDNA technology, completed a Phase 2b trial and obtained fast track designation by the FDA for treating pain associated with knee OA [152]. These viral and non-viral technologies are promising approaches for preventing and regenerating tissue in the management of OA and synovial inflammation.

## 5 Discussion

The clinical and research landscape of knee OA, particularly in relation to synovitis, is shaped by persistent challenges and evolving paradigms. A central controversy, the cause-versus-consequence debate, carries important clinical implications. Synovial inflammation can act both as a trigger and as a consequence of joint damage. Evidence increasingly demonstrates the role of systemic metabolic dysfunction, including obesity and dyslipidaemia, in driving synovitis through metaflammation, while abnormal mechanical loading contributes via mechanoflammation. Together, these mechanisms form a self-reinforcing cycle that accelerates joint degeneration and complicates therapeutic decision-making.

From a clinical standpoint, several questions arise:

### 5.1 Is synovial inflammation a cause or a consequence of OA, and why does it matter clinically?

The distinction between cause and consequence remains unresolved, yet it is clinically relevant. In early disease, subchondral bone changes and abnormal mechanical stress may initiate synovitis, whereas in established OA, inflammation more often arises as a secondary response to structural damage [43,153,154]. Metabolic dysfunction further complicates this relationship, acting both as a driver and an amplifier of inflammation. Clinically, this duality supports the use of early detection strategies, such as MRI, to identify individuals at high risk before irreversible cartilage loss occurs. Interventions targeting the crosstalk between bone, synovium, and cartilage may be most effective in early OA, while biomechanical management remains essential at later stages [155]. Recognition of a metabolic OA phenotype emphasises the importance of metabolic risk screening and tailored treatment approaches [156,157].

### 5.2 Is there a clinically relevant metabolic phenotype of OA?

Current evidence supports the existence of a “metabolic OA” phenotype that may respond differently to pharmacological and lifestyle interventions than mechanically driven OA [22,27,156,[158], [159], [160]]. This recognition suggests that metabolic screening and cardiovascular risk management could become integral components of OA care. The key question is whether obesity and dyslipidaemia initiate OA or primarily amplify pre-existing degeneration. Recent cohort studies indicate that metabolic disturbances often precede radiographic disease, but the bidirectional interplay of inflammation and altered biomechanics complicates causal interpretation [161]. Clinically, acknowledging a metabolic phenotype provides a rationale for tailored management strategies that extend beyond the joint to address systemic risk factors [156,157].

### 5.3 What is the clinical promise of biomarkers in knee OA?

Biomarkers are reshaping the approach to knee OA by shifting the focus from static structural assessment toward dynamic measures of disease activity. Circulating markers such as MMP-3, MMP-13, NTX-I, PIIINP, ghrelin, and IPFP-derived adipokines provide insight into the biological drivers of synovitis, cartilage degradation, and pain [54,162,163]. Their correlation with imaging features, symptom severity, and disease progression supports their potential role in patient stratification and disease monitoring. Certain biomarkers also appear predictive of treatment response, such as chondroitin sulphate, tenascin-C, and aggrecan for HA injections, or white blood cell counts for corticosteroid response [56].

Advanced imaging, particularly MRI, complements these biochemical tools by detecting early synovitis, subclinical cartilage damage, and bone alterations [[59], [60], [61],^164^]. The integration of AI into imaging analysis and emerging brain imaging studies provides new perspectives on pain mechanisms and prognosis [165]. Importantly, these developments also draw attention to the limitations of relying on pain alone as a marker of OA, since many patients remain asymptomatic despite advanced disease, whereas others report pain in the absence of major structural changes [63,166,167]. Clinically, this calls for multidimensional assessment strategies that combine symptoms, imaging, and biomarkers to characterise disease activity and guide care better.

### 5.4 Can molecular endotypes transform OA treatment?

The identification of molecular endotypes represents a critical step toward precision medicine in OA. By moving beyond symptom- and radiograph-based classifications, clinicians can align treatment with the underlying biological mechanisms, whether inflammatory, fibrotic, structural, or metabolic. This approach enhances therapeutic effectiveness, improves patient stratification, and strengthens prognostic accuracy [[57], [58], [59],^61^]. The inflammatory endotype, characterised by high synovial cytokine activity and macrophage-driven inflammation, is now well recognised and represents a promising therapeutic target. Endotype-based treatment design also stresses the importance of synovial fibroblasts and macrophages as potential targets for future therapies, offering opportunities to intervene more effectively in the mechanisms driving disease progression [52].

### 5.5 How can we move beyond symptomatic relief to target inflammatory pathways?

Despite advances in understanding, current treatment for knee OA synovitis remains largely symptomatic. NSAIDs remain first-line pharmacological agents but carry long-term risks. Alternatives such as paracetamol, colchicine, and diacerein have variable efficacy, while IA corticosteroids, HA, and PRP provide short-to medium-term relief with inconsistent long-term benefits.

Emerging therapies, however, signal a shift toward targeting inflammatory pathways. Inflammasome inhibitors, TNF-α and IL-1β blockers, and methotrexate offer promise in modulating systemic and local inflammation. IA innovations such as iPAAG and carboxymethyl-chitosan may provide durable mechanical and possibly anti-inflammatory benefits. Minimally invasive approaches such as genicular artery embolization and cryoneurolysis further expand interventional options. Together, these developments support a more personalised, mechanism-based approach to OA care, where treatment is guided by phenotype, biomarkers, and imaging rather than symptoms alone [52].

### 5.6 What is the therapeutic potential of synovium-targeted gene therapies?

Gene and cell-based therapies are emerging as a paradigm shift, with the synovium positioned as both a delivery site and a therapeutic target [168]. Strategies such as Allocetra, PLX-PAD, and PCRX-201 aim to directly modulate synovial inflammation, while mesenchymal stem cell therapies and TissueGene-C indirectly improve the joint environment through immunomodulation and regenerative effects. Viral and non-viral vectors now allow the selective delivery of anti-inflammatory or regenerative genes to synovial tissue, offering the possibility of long-lasting IA treatments that reduce the need for repeated injections [169].

Clinically, these approaches could move OA management beyond symptom relief toward actual disease modification. By directly addressing synovitis, gene and cell therapies have the potential to alter disease trajectory, particularly in metabolically driven or inflammatory phenotypes [168]. Nevertheless, challenges remain, including vector immunogenicity, age-related transduction efficiency, production costs, and clinical validation. Ongoing research into dosing strategies, patient stratification, and endotype-driven trial design will be crucial to translating these therapies into practice.

### 5.7 Future directions

This evolving understanding of OA necessitates a shift away from traditional symptom- and radiograph-based models toward the early identification of high-risk individuals using imaging, biomarkers, and molecular profiling. The recognition of distinct OA phenotypes and endotypes reinforces the need for tailored interventions that target both local and systemic drivers of inflammation [52,53]. At present, NSAIDs and IA corticosteroids remain the mainstay of care, but the future of OA management lies in mechanism-based and personalised approaches that integrate metabolic screening, molecular profiling, and regenerative therapies.

## 6 Conclusion

In summary, synovitis is not merely a secondary feature of OA but a central and modifiable driver of disease progression. Advancing the management of knee OA synovitis requires progress in biomarker discovery, imaging technologies, and targeted therapeutic development, together with the broader adoption of precision medicine frameworks. Meeting these challenges offers the potential not only to improve symptom control but also to slow or halt structural progression, ultimately reshaping outcomes for patients with OA.

## Declaration of Generative AI in scientific writing

The authors declare that generative AI and AI-assisted technologies were not used for drafting the text and preparing figures in this manuscript.

## Author contribution

All authors contributed equally to conceptualization, investigation, methodology, project administration, resources, supervision, validation, visualization, and writing of the original draft, as well as to reviewing and editing the final version of the manuscript, and all authors agreed to its submission. AM also contributed to funding acquisition. No roles related to software, data curation, or formal analysis were required for this manuscript.

## Funding statement

Contura International Limited (United Kingdom) provided financial support to write the manuscript.

## Declaration of competing interest

AM served as former President of the Osteoarthritis Research Society International (OARSI) (May 2019–May 2022) and a member of the OARSI Board of Directors from 2013 to 2022. He currently serves as an advisor for the World Health Organization Collaborating Center for Public Health Aspects of Musculoskeletal Health and Aging, Member-at-Large on the Steering Committee Osteoarthritis Action Alliance, and Member of the Scientific Advisory Board of the European Society for Clinical and Economic Aspects of Osteoporosis, Osteoarthritis and Musculoskeletal Diseases (ESCEO).

AM provides scientific consulting on the advisory boards for HALEON (Global Pain Faculty, Naturals Advisory Board), Sanofi, Sanofi Consumer Healthcare (Opella Healthcare), Kolon TissueGene, Enlivex, Pacira BioSciences, Contura, Chondrometrics, Aptissen SA, Synartro AB, Contura AB, ICM (South Korea), Kangstem, Peptinov, Pluri, Chondropeptix and the California Institute for Regenerative Medicine (CIRM), California's Stem Cell Agency.

AM also acknowledges consortium funding from 10.13039/100018693Horizon Europe (PROTO - Advanced Personalised Therapies for Osteoarthritis – TACKLING INFLAMMATION TO IMPROVE PATIENT OUTCOMES, Grant agreement ID:101095635, https://cordis.europa.eu/project/id/101095635; ENgineered CArtilage from Nose for the Treatment of Osteoarthritis (ENCANTO), Grant agreement ID: 101137315, https://cordis.europa.eu/project/id/101137315); the European Structural and Social Funds through the 10.13039/501100004504Research Council of Lithuania (10.13039/501100004504Lietuvos Mokslo Taryba), according to the Program Attracting Foreign Researchers for Research Implementation (Grant No. 01.2.2-LMT-K-718-02-0022) plus networking grant support from the 10.13039/501100000921European Cooperation in Science and Technology (10.13039/501100000921COST) Association, Action CA21110 – Building an open European Network on OsteoArthritis research (NetwOArk; https://www.cost.eu/actions/CA21110/).

FC served as former Chair of the Spanish Society of Rheumatology Osteoarthritis Task Force (May 2020–May 2024), Secretary of the Spanish Society of Rheumatology Osteoarthritis Task Force (May 2018–May 2020). FC provided scientific consulting on the advisory boards for Pfizer, IBSA and Grünenthal.

SK served as former Grant Committee Chair and Board Member of the British Association of Sports Medicine. SK serves as a Codirector of Oxford Medicine and Research LTD. He currently provided scientific consulting on the advisory boards for Contura.

SPR received professional fees from Contura for supporting the writing and editing of this manuscript.

O.G. holds a position as A1 Staff Personnel (I3SNS Stable Researcher) at Xunta de Galicia (Servizo Galego de Saude (SERGAS). O.G. is a member of the RICORS Program, RD24/0007/0031, through ISCIII and FEDER European Union and European Commission. The research of O.G. (PI23/00289) is financially supported by ISCIII and FEDER European Union and European Commission. O.G. is a member of the COST action CA21110 (Building a European Network on Osteoarthritis (NETWOARK)), funded by the European Union and European Commission under the European Cooperation in science and technology program (COST). O.G. received a grant from GEER (Sociedad Española de Columna Vertebral) Beca GEER 2023. O.G. is the beneficiary of a grant funded by Xunta de Galicia, Consellería de Educación, Universidade e Formación Profesional and Consellería de Economía, Emprego e Industria (GAIN) (GPC IN607B-2025/03). O.G. received a grant from Fundación Mutua Madrileña AP-21009/2025. The funders were not involved in study design, data collection and analysis, decision to publish, or manuscript preparation.

RL has no conflicts of interest to declare.
